# A Case Series of Complex Recalcitrant Wounds Treated with Epidermal Grafts Harvested from an Automated Device

**DOI:** 10.7759/cureus.853

**Published:** 2016-10-30

**Authors:** Stephen S Cai, Arvind U Gowda, Karan Chopra, Rachel Waldman, Ronald P Silverman, Yvonne M Rasko

**Affiliations:** 1 Division of Plastic Surgery, University of Maryland School of Medicine; 2 Department of Plastic and Reconstructive Surgery, Yale School of Medicine; 3 Department of Plastic and Reconstructive Surgery, The Johns Hopkins Hospital

**Keywords:** epidermal grafting, suction blister epidermal grafting, complex wounds, cellutome

## Abstract

Introduction: Epidermal grafting has several advantages over full-thickness or split-thickness grafts in the treatment of complex non-healing wounds. These include the low risk of donor site complications, minimal patient discomfort, and abstention from the operating room. Traditionally, the lack of reliable epidermal harvesting techniques has limited its clinical utilization. The development of an automated suction blister epidermal graft (SBEG) harvesting device may facilitate clinical utilization of this technique. The authors present a case series of multimorbid patients who were poor surgical candidates and were treated with this technique.

Methods: A retrospective review of all patients treated with CelluTome™​ Epidermal Harvesting System (KCI, an Acelity company, San Antonio, TX) prior to May 2016 at our institution was conducted.

Results: A total of 12 patients underwent 14 epidermal grafting procedures. Multiple comorbidities were identified, including smoking (33%), immunosuppression by immunotherapy or steroids (25%), chronic venous insufficiency (25%), diabetes mellitus (25%), malignancy (25%), polysubstance abuse (17%), HIV/AIDS (17%), and peripheral artery disease (8%). Among the two acute wounds (≤ 3 months) and 10 chronic wounds, the average wound size was 49.1 cm^2^ (± 77.6 cm^2^) and the median wound duration was 5.7 months (interquartile range: 4.1 - 15.8 months) before SBEG was attempted. These complex wounds had failed prior therapies, such as local wound care (100%), incision and drainage (58%), vacuum-assisted closure (33%), split-thickness skin graft (16%), and hyperbaric oxygen (8%). Following the procedure, all donor sites healed within one week. Three patients were lost to follow-up. Of the remaining nine patients, four patients had complete resolution of their wounds at a median follow-up of 13.1 weeks (interquartile range: 6.8-17.3 weeks). Among those with partial resolutions, the average wound size was 4.2 cm^2^ (± 2.1 cm^2^) with an average wound reduction of 79% (± 23%). No donor or recipient site complications were observed.

Conclusions: The automated SBEG harvesting device is an effective and safe option for treating complex non-healing wounds in multimorbid patients who may be poor surgical candidates. This procedure demonstrates minimal contraindications to its use and donor or recipient site complications.

## Introduction

Complex recalcitrant wounds are notoriously difficult to manage because they do not follow ordinary regeneration and repair. In the United States alone, approximately 3 to 6 million patients suffer from such wounds and an increasing number of patients are treated insufficiently over protracted courses, costing an estimated $5 to $10 billion each year [1]. The use of split-thickness or full thickness skin grafts offers a reasonable solution; however, it is limited by donor site availability, poor healing at the donor site, and painful skin harvesting procedures that require general anesthesia and a trip to the operating room [2-3]. Alternatively, artificially engineered skin and allograft or xenograft provide rapid but temporary coverage and are often prohibitively expensive with unreliable outcomes.

First pioneered by Kiistala and Mustakallio in 1964, suction blister epidermal grafting (SBEG) offers a readily available, non-immunogenic, and non-invasive method of wound healing [4]. Early studies reported success in treating skin conditions, such as leukoderma, hypopigmentation, and refractory or stable vitiligo, to replenish melanocytes as well as chronic leg ulcers and non-healing wounds after excision of skin cancer [5-10].

There are several notable advantages to autologous epidermal grafts. Harvesting the epidermis is relatively painless and can be performed without anesthesia. Additionally, the minimally invasive depth of epidermal grafting is believed to cause only a minor inflammatory response and the donor site is expected to heal in three to four days [11-12]. In contrast, alternative methods of skin grafting are likely to incur operating room expenses, require general anesthesia, and may result in donor site morbidity, which all serve to limit its use in patients who are poor surgical candidates. Conventional methods of obtaining SBEG involve a free hand-blade or syringe suction to create epidermal micro-blisters, which proved to be technically challenging and particularly time-consuming. Thus, despite its obvious advantages, SBEG failed to achieve widespread use due to lack of a reliable harvesting technique.

An automated epidermal harvesting system, CelluTome™​ Epidermal Harvesting System (KCI, an Acelity company, San Antonio, TX), recently became commercially available. This automated system combined heat and suction to produce epidermal blisters that can be harvested and transferred to a recipient site. Current literature on the CelluTome™​ is limited. The purpose of this paper is to present a single-site experience with CelluTome™​ in a large urban hospital setting and evaluate its potential in the treatment of complex recalcitrant wounds in a multimorbid patient population.

## Materials and methods

### Data collection

This is a retrospectively reviewed case series with approval by the University of Maryland, Baltimore Institutional Review Board (approval HP-00067976). Informed patient consent was obtained at the time of treatment.

Patients were identified through the institutional electronic medical records. Inclusion criteria required patients to undergo one or multiple SBEG using the CelluTome™ Epidermal Harvesting System prior to May 2016. No exclusion criteria were identified.

Medical records of the eligible patients were reviewed. Patient demographics, including age, sex, and comorbidities that impair wound healing, were obtained. Clinical data included wound type or etiology, wound location, and prior wound care. Operative information was collected that included initial wound size, donor site, final wound size at follow-up, and time to follow-up. Primary outcome focused on the degree of wound closure. Complete resolution was defined as the complete union of wound edge with viable epithelialization and neovascularization of the graft. In cases of partial resolution, reduction in wound size was calculated as a percent of the initial wound size.

### Harvesting technique

**Figure 1:**
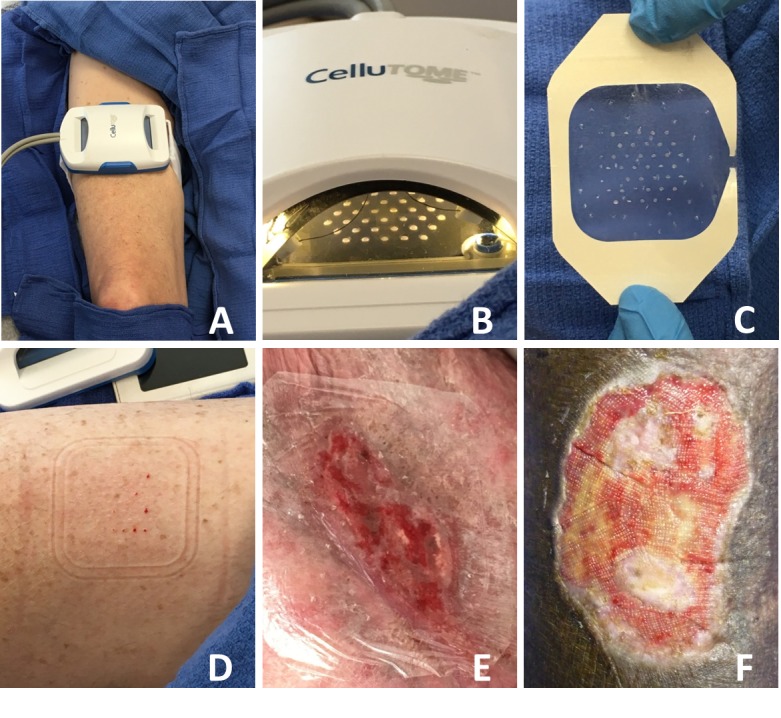
Epidermal Autologous Skin Graft Harvested Using the CelluTome™ Epidermal Harvesting System. (A) The device is securely fastened to the patient’s right thigh and suction is applied. (B) When sufficient microblisters have formed, the epidermal graft is harvested onto a (C) perforated TegadermTM film. (D) Immediately post-harvest, the donor site demonstrated minimal bleeding. (E) The harvested graft is directly applied to the recipient site and covered with an appropriate dressing. (F) Islands of epithelialization can be observed at four weeks postoperatively. ​

Epidermal grafts were procured with the CelluTome™ Epidermal Harvesting System, taking into consideration the warnings, precautions, and instructions listed in the device manual. An appropriately sized donor site on the thigh was prepared by removing the hair and rigorous cleaning with alcohol. The harvester plate was then centered on the site, ensuring complete contact with skin, and secured (Figure 1A). The vacuum head was then attached to the harvester and the device was turned on to begin the micro-blister raising process (-400 to -500 mmHg and 39º to 41º C). The development of epidermal micro-blisters can be observed through a window on the vacuum head (Figure 1B). Once blisters were fully formed (30 to 45 minutes), vacuum head was removed and a perforated Tegaderm^TM^ film was applied to the harvester to capture the epidermal grafts and allow transfer (Figure 1C). Perforation of the film allowed drainage of fluid when cutting the micro-blisters. The donor site was then covered with a Tegaderm^TM^ transparent film dressing and a dry dressing (Figure 1D).

The recipient site was meticulously scrubbed to remove any debris. Tincture of benzoin was applied to the surrounding skin for better adhesion. The epidermal grafts carried by the perforated Tegaderm^TM^ were directly applied to the recipient site and then covered with an appropriate pressure dressing (Figure 1E). Negative pressure wound therapy was often applied for one week to eliminate residual moisture and allow optimal apposition of the graft in areas of the body where a compression dressing was not easily placed, such as the groin and axilla. Evidence of epithelialization was observed within the wound bed, with islands of graft take visible (Figure 1F).

## Results

A retrospective review identified a total of 12 patients, six females and six males, who underwent SBEG procedures using the CelluTome™​ Epidermal Harvesting System (Table 1). Several comorbidities that impaired wound healing were present, including smoking (33%), immunosuppression by immunotherapy or steroids (25%), chronic venous insufficiency (25%), diabetes mellitus (25%), malignancy (25%), polysubstance abuse (17%), HIV/AIDS (17%), and peripheral artery disease (8%). There were two acute wounds (defined as ≤ 3 months in duration) and 10 chronic wounds. The average wound size was 49.1 cm2 (± 77.6 cm2). The median wound duration was 5.7 months (interquartile range: 4.1 - 15.8 months) before SBEG was attempted. In all cases, the wounds failed to heal with local wound therapy (100%). Most have failed one or more combinations of incision and drainage (58%), vacuum-assisted closure (VAC) (33%), split-thickness skin graft (16%), and hyperbaric oxygen (8%). Etiologies of these wounds are multifaceted but can be largely classified into incisional (renal transplant, cancer resection, and fasciotomy) (33%), vascular (vascular graft failure and venous ulcer) (25%), inflammatory (hidradenitis) (25%), and orthopedic (infected hardware and open reduction internal fixation) (17%).

**Table 1:** Patient Demographic and Clinical Characteristics AKA - above knee amputation; CA – cancer; CVI - chronic venous insufficiency; DM - diabetes mellitus; HBO - hyperbaric oxygen; HIV - human immunodeficiency virus; IMSP - immunosuppressed: immunotherapy or chronic steroid use; IND -incision and drainage; LWC - local wound care; ORIF - open reduction internal fixation; PAD - peripheral artery disease; RLE - right lower extremity; SA - polysubstance abuse; SM – smoker; STSG - split-thickness skin graft; VAC - vacuum-assisted closure

	Age (y)	Sex	Comorbidities	Wound Type	Location	Prior Treatment	Wound Duration (mos)	
1	13	M	None	Abscess	Right Thigh	LWC, VAC		1.1
2	32	F	DM	Hidradenitis	L Axilla	LWC, IND		61.0
3	46	M	SM	Hidradenitis	R Buttock	LWC, IND		25.9
4	54	F	HIV, CVI, SA	Venous Ulcer	RLE	LWC, IND, STSG		43.5
5	56	F	DM, IMSP	Renal Transplant	Abdomen	LWC		3.8
6	58	M	IMSP	Fasciotomy	L Groin	LWC, VAC		5.3
7	59	M	IMSP, SM, SA	Infected Hardware	L Ankle	LWC, IND		1.0
8	63	M	Basal cell CA, SM	CA Resection	L shoulder	LWC, IND, VAC		4.3
9	65	M	HIV, DM	ORIF	L Ankle	LWC		12.5
10	76	F	Colon CA, CVI, SM	Vascular Graft Failure	L AKA Stump	LWC, IND, STSG, HBO		4.5
11	81	F	Ovarian CA	CA Resection	Abdomen	LWC, VAC		6.2
12	82	F	CVI, PAD	Vascular Graft Failure	RLE	LWC, IND, VAC		6.2

The SBEG procedure was performed on 14 occasions (one patient underwent three consecutive procedures) (Table 2). Donor sites were chosen on alternating thighs when more than one graft was required. Following the procedure, all donor sites healed within one week. Three patients were lost to follow-up: one as a result of non-compliance and two due to changes in clinical management, such that wound care was no longer required. The remaining nine patients were followed for a median follow-up of 13.1 weeks (interquartile range: 6.8 - 17.3 weeks). Four patients (44%) had complete resolution of their wounds. Among those with partial resolutions, the average wound size was 4.2 cm2 (± 2.1 cm2) with an average wound reduction of 79% (± 23%). No donor or recipient site complication was observed in any of the patients.

**Table 2:** Patient Treatment and Outcomes CR - complete resolution; LTFU - lost to follow-up; PR - partial resolution

	Initial Wound Size (cm)	Donor Site Healing (wks)	Time to Follow-up (wks)	Final Wound Size (cm)	Wound Reduction (%)	Outcome
1	10.5 x 2.0	< 1	2.7	2 x 2	81	PR
2	4.8 x 5.0	1	16.7	1 x 1	96	PR
3	5.0 x 3.5	1	12.9	2.5 x 1.5	79	PR
4	5.0 x 5.5	1	10	5 x 4.2	24	
	6.8 x 5.2	1	16	6.5 x 4.5	17	
	6.5 x 4.5	N/A	N/A	N/A	N/A	LTFU^1^
5	5.0 x 4.5	1	18	0 x 0	100	CR
6	15.3 x 4.0	1	13.1	0 x 0	100	CR
7	4.8 x 1.8	1	N/A	N/A	N/A	LTFU^2^
8	5.0 x 3.0	1	6.1	0 x 0	100	CR
9	4.2 x 2.5	<1	7.6	3.1 x 2	41	PR
10	2.5 x 2.0	1	5.1	0 x 0	100	CR
11	5.0 x 4.0	N/A	N/A	N/A	N/A	LTFU^3^
12	14.0 x 20.0	1	19	4 x 1.5	98	PR
^1^ SBEG was attempted on three separate occasions; the patient did not return for follow-up after her third procedure.						
^2^ The wound was re-explored after four days and orthopedic hardware was removed in the operating room.						
^3^ Patient deteriorated clinically after two days and warranted ICU admission. The patient declined further treatment.						

### Case 1: 58-year-old man with an extensive surgical history who refused to undergo general anesthesia and another operation

**Figure 2:**
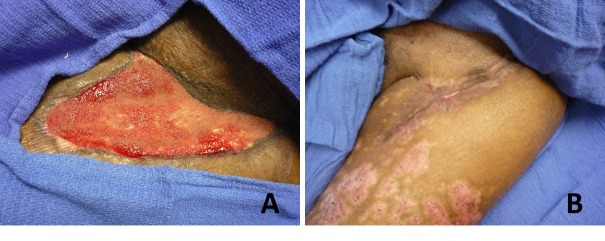
A 58-Year-Old Male with an Extensive Surgical History Who Declined to Undergo Another Operation Under General Anesthesia. (A) Wound measured 15.3 x 4 cm on presentation. (B) Epidermal graft is performed and complete resolution of the wound is achieved at 13 weeks without complications.

The patient is a 58-year-old gentleman with multiple comorbid conditions including hypertension, chronic thromboembolic disease, and pulmonary hypertension with recurrent pulmonary emboli status-post thromboembolectomy complicated by coagulopathic bleeding. In the two months following his thromboembolectomy, the patient underwent 11 operations, including cholecystectomy, abdominal exploratory laparotomy, tracheostomy, left lower extremity fasciotomy, and systemic venoarterial extracorporeal membrane oxygenation cannulation. The Plastic Surgery Service was consulted to assist with the further care of the left groin fasciotomy wound secondary to compartment syndrome that had failed to heal after five months of local wound care and negative pressure wound therapy. The patient refused to undergo another operation under general anesthesia due to the multiple complications over a prolonged hospital course and opted for a less invasive intervention with epidermal skin grafting. On examination, the wound bed was clean with no evidence of infection and measured 15.3 x 4 cm (Figure 2A). Epidermal skin grafting was performed with two 5 x 5 cm skin grafts harvested from the right thigh and application of a wound VAC for one week. Complete closure of the wound was achieved at 13 weeks with no complications (Figure 2B).

### Case 2: 63-year-old current smoker of over 30 pack-years status-post radical resection and external radiation for basal cell carcinoma of the left shoulder

**Figure 3:**
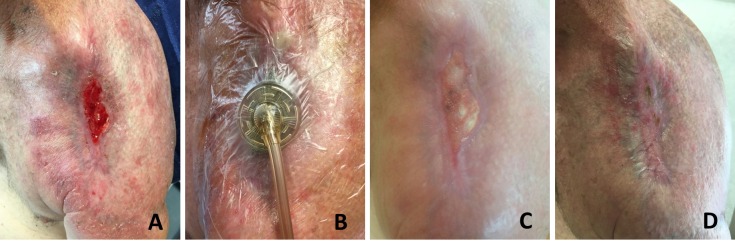
63-Year-Old Current Smoker of Over 30 Pack-Years Status-Post Radical Resection and External Radiation for Basal Cell Carcinoma of the Left Shoulder. (A) Wound measured 5 x 3 cm. (B) Epidermal graft is performed and vacuum-assisted closure is applied for one week postoperatively. (C) Islands of epithelialization can be seen within the wound bed two weeks from the index operation. (D) Complete resolution of the wound is achieved at six weeks without complications.

The patient is a 63-year-old gentleman with a history of longstanding neglected basal cell carcinoma that eroded his left shoulder status-post radical resection and radiation therapy who presented with pain, erythema, swelling, fever, and purulent drainage from his left shoulder concerning for osteomyelitis and surgical site infection. He has over 30 pack-years of smoking history and has no plans of quitting despite extensive discussion. Resection of the clavicle and proximal humerus was performed. Wound closure was attempted with adjacent tissue transfer and advancement of the previous latissimus dorsi myocutaneous flap. Negative pressure wound therapy was applied to the residual wound. For the next six months, the patient continued to smoke as the wound failed to heal and was primarily managed by local wound care and home negative pressure wound therapy. Skin graft options were discussed with the patient. He was a poor candidate for surgery, given his smoking history and poor compliance with follow-up, and elected to undergo epidermal grafting. Epidermal skin grafting was performed on a clean, healthy wound measuring 5.0 x 3.0 cm over the left shoulder (Figure 3A). Wound VAC was applied to the wound for the following week (Figure 3B). Islands of epithelialization could be seen within the wound bed two weeks following the index operation (Figure 3C). Complete closure of the wound was achieved at six weeks with no complications (Figure 3D).

## Discussion

The use of SBEG on chronic, non-healing wounds is not a novel idea. Hentzer and Kobayasi first discussed the treatment of chronic leg ulcers using epidermal grafts in 1975. A number of studies have reported success in treating skin conditions, including leukoderma, hypopigmentation and vitiligo, and chronic non-healing wounds, such as venous leg ulcers and diabetic foot ulcers [13-15]. However, the tedious harvesting process using a free blade or syringe-suction has stifled the widespread use of this technique. The advent of an automated harvester offered a reliable, reproducible, and efficient alternative. The present study discussed the CelluTome™​ Epidermal Harvesting System in multimorbid patients with recalcitrant wounds that have failed prior wound therapy in a large U.S. urban hospital setting.

The usefulness of CelluTome™​ Epidermal Harvesting System was first demonstrated in Port-au-Prince, Haiti where insufficient resources and lack of clinical training limited their options in wound care [12]. Similarly, automated SBEG also proved useful when split-thickness skin graft (STSG) was contraindicated out of concern for poor wound healing at the donor site, such as the case in pyoderma gangrenosum [11]. The likely explanation is that the minimally invasive depth of SBEG, specifically cleavage of the lamina lucida with preserved epidermal ultrastructure, induces little inflammatory response, which results in expedited healing [16]. Subsequent reports on abrasion and burn due to trauma, heat burn to a radiated breast, a scalp melanoma excision site, a wound created by tattoo removal, and keloid reconstructions all described a partial or complete resolution of the wounds [17-19]. More recently, several case series, including a large prospective trial from the UK, has shown promising results in an outpatient setting [20-22]. The cost-effectiveness of SBEG cannot be overstated and estimated to be £431 per procedure, approximately one-third the cost of STSG [23]. As a result of the aforementioned studies, multicenter randomized controlled clinical trials are now in progress in the US and the UK to evaluate the effectiveness of the CelluTome™​ Epidermal Harvesting System in the treatment of venous leg ulcers and other wounds [24-25].

Molecular studies on SBEG using CelluTome™​ showed that, with the automated system, epidermal micrografts formed at the dermal-epidermal junction with high expressions of Ki67 nuclear protein – an indicator of mitotic activity. The grafts also secreted a host of growth factors essential to wound healing, including platelet-derived growth factor, vascular endothelial growth factor, and granulocyte colony-stimulating factor [26-27]. These findings are consistent with our observation that when the epidermal graft is transplanted, wound healing occurred simultaneously from within the wound bed and from the margins. These islands of re-epithelialization eventually merged to form a confluent structure, which suggests a unique molecular mechanism of epidermal grafts and warrants further investigation.

In treatment of complex wounds, automated SBEG has several clear advantages over full-thickness or split-thickness skin grafts: 1) it is a technically straightforward procedure that can be performed in an outpatient setting without the need for general anesthesia or operating room time; 2) there is minimal discomfort to the patient peri- and postoperatively; and 3) there is rapid donor site healing without complications or scarring. Our study reaffirmed these observations in multimorbid patients that would typically have wound healing difficulties. All donor sites on the patients’ thighs healed within one week of the procedure. Donor or recipient site complications, such as bleeding or infection, were not observed in any of the cases.

Results of our study revealed several novel findings. Firstly, the procedure can be repeated if necessary. One patient received three SBEGs in the span of eight months. Reduction of the wound was observed after each application. The senior author believed that it was in the best interest of the patient to try again despite compliance issues. No donor or recipient site complication was observed during any of the procedures. In effect, SBEG can be attempted with little or no repercussion. Secondly, in terms of technique, elimination of moisture is a priority in our experience. Our use of the perforated Tegaderm^TM^ film and VAC postoperatively worked in concert to reduce moisture on the wound surface and likely improved the chances of graft take and wound healing. Lastly, multiple donor sites did not cause significant morbidity. Several grafts were required as a result of the extensive wounds some patients possessed. Donor sites were chosen on alternating thighs. However, in cases where a third donor site was necessary in an adjacent area on the ipsilateral thigh, no significant complication occurred, and all donor sites healed within one week. 

As with any retrospective study, selection bias is the main limitation. In dealing with complex wounds, patient selection is an integral component of a successful outcome. Patients with comorbidities not suitable for the operating room, wound healing issues, and compliance concerns are poor candidates for traditional skin grafts. The autologous epidermal grafts effectively circumvent these problems and present an attractive alternative. The creation of an automated SBEG harvesting technique further simplified the procedure and minimized postoperative complications. Although our study has shown success in a variety of patients, identification of the ideal patient population may be of interest in follow-up studies. So far, we have yet to identify any contraindications to the application of SBEG.

## Conclusions

In conclusion, SBEG harvested using the CelluTome™​ Epidermal Harvesting System successfully resolved or reduced the wounds of various etiologies in a patient population that included those with multiple comorbidities that impair wound healing, those who have failed prior attempts at wound closure, and those who are unsuited for surgical correction under general anesthesia. Application of the autologous epidermal grafts incurred no additional complications at the donor or recipient site and provided a viable solution to their wounds, even in those who failed SBEG previously.

## References

[1] Werdin F, Tennenhaus M, Schaller HE, Rennekampff HO (2009). Evidence-based management strategies for treatment of chronic wounds. Eplasty.

[2] Biswas A, Bharara M, Hurst C, Armstrong DG, Rilo H (2010). The micrograft concept for wound healing: strategies and applications. J Diabetes Sci Technol.

[3] Serena TE (2015). Use of epidermal grafts in wounds: a review of an automated epidermal harvesting system. J Wound Care.

[4] Kiistala U, Mustakallio KK (1967). Dermo-epidermal separation with suction. Electron microscopic and histochemical study of initial events of blistering on human skin. J Invest Dermatol.

[5] Gupta S, Jain VK, Saraswat PK (1999). Suction blister epidermal grafting versus punch skin grafting in recalcitrant and stable vitiligo. Dermatol Surg.

[6] Njoo MD, Westerhof W, Bos JD, Bossuyt PM (1998). A systematic review of autologous transplantation methods in vitiligo. Arch Dermatol.

[7] Kim HY, Kang KY (1999). Epidermal grafts for treatment of stable and progressive vitiligo. J Am Acad Dermatol.

[8] Budania A, Parsad D, Kanwar AJ, Dogra S (2012). Comparison between autologous noncultured epidermal cell suspension and suction blister epidermal grafting in stable vitiligo: a randomized study. Br J Dermatol.

[9] Parbhoo AV, Simpson MT (2014). Suction blister skin grafting--a modern application. Br J Oral Maxillofac Surg.

[10] Costanzo U, Streit M, Braathen LR (2008). Autologous suction blister grafting for chronic leg ulcers. J Eur Acad Dermatol Venereol.

[11] Richmond NA, Lamel SA, Braun LR, Vivas AC, Serena T, Kirsner RS (2014). Epidermal grafting using a novel suction blister-harvesting system for the treatment of pyoderma gangrenosum. JAMA Dermatol.

[12] Serena T, Francius A, Taylor C, MacDonald J (2015). Use of a novel epidermal harvesting system in resource-poor countries. Adv Skin Wound Care.

[13] Hentzer B, Kobayasi T (1975). Suction blister transplantation for leg ulcers. Acta Derm Venereol.

[14] Hefton JM, Madden MR, Finkelstein JL, Shires GT (1983). Grafting of burn patients with allografts of cultured epidermal cells. Lancet.

[15] Hefton JM, Caldwell D, Biozes DG, Balin AK, Carter DM (1986). Grafting of skin ulcers with cultured autologous epidermal cells. J Am Acad Dermatol.

[16] Willsteed EM, Bhogal BS, Das A, Bekir SS, Wojnarowska F, Black MM, Mckee PH (1991). An ultrastructural comparison of dermo-epidermal separation techniques. J Cutan Pathol.

[17] Howarth AL, Bell BE, Peterson WC, Renz EM, King BT, Chan RK (2015). A novel approach to graft loss in burn using the CelluTome™ epidermal harvesting system for spot grafting: a case report. Burns.

[18] Gabriel A, Sobota RV, Champaneria M (2014). Initial experience with a new epidermal harvesting system: overview of epidermal grafting and case series. Surg Technol Int.

[19] Nguyen KT, Shikowitz L, Kasabian AK, Bastidas N (2016). A novel approach to keloid reconstruction with bilaminar dermal substitute and epidermal skin grafting. Plast Reconstr Surg.

[20] Hachach-Haram N, Bystrzonowski N, Kanapathy M, Smith O, Harding K, Mosahebi A, Richards T (2016). A prospective, multicentre study on the use of epidermal grafts to optimise outpatient wound management. Int Wound J.

[21] Fearmonti RM (2016). Efficacy of epidermal skin grafts over complex, chronic wounds in patients with multiple comorbidities. Wounds.

[22] Bhatia A (2016). Epidermal skin grafting in patients with complex wounds: a case series. J Wound Care.

[23] Smith OJ, Edmondson SJ, Bystrzonowski N, Hachach-Haram N, Kanapathy M, Richards T, Mosahebi A (2016). The CelluTome epidermal graft-harvesting system: a patient-reported outcome measure and cost evaluation study. Int Wound J.

[24] Serena T (2016). Clinical Trial to Evaluate Blister Graft Utilizing a Novel Harvesting Device for Treatment of Venous Leg Ulcers (Cellutome). ClinicalTrials.gov.

[25] Kanapathy M, Hachach-Haram N, Bystrzonowski N, Harding K, Mosahebi A, Richards T (2016). Epidermal grafting versus split-thickness skin grafting for wound healing (EPIGRAAFT): study protocol for a randomised controlled trial. Trials.

[26] Osborne SN, Schmidt MA, Derrick K, Harper JR (2015). Epidermal micrografts produced via an automated and minimally invasive tool form at the dermal/epidermal junction and contain proliferative cells that secrete wound healing growth factors. Adv Skin Wound Care.

[27] Osborne SN, Schmidt MA, Harper JR (2016). An automated and minimally invasive tool for generating autologous viable epidermal micrografts. Adv Skin Wound Care.

